# Harmful newborn cord care practices and associated factors among mothers who gave birth in the last six months in Chencha town, Southern Ethiopia: a mixed-methods study

**DOI:** 10.3389/fped.2024.1492222

**Published:** 2025-01-28

**Authors:** Misgana Seifu, Sultan Hassen, Mekdim Kassa, Yosef Haile, Zeleke Girma, Temesgen Mohammed Toma, Agune Ashole, Mintesinot Melka Gujo, Wondimagegn Taye Dema, Aleme Mekuriya, Endashaw Shibru

**Affiliations:** ^1^ Chencha Woreda Health Office, Chencha, Gamo Zone, Ethiopia; ^2^ College of Medicine and Health Sciences, School of Public Health, Arba Minch University, Arba Minch, Ethiopia; ^3^ South Ethiopia Region Health Bureau Public Health Institute, Regional Data Management Center, Jinka, Ethiopia; ^4^ South Ethiopia Region Health Bureau Public Health Institute, Jinka, Ethiopia; ^5^ Arbaminch College of Health Sciences, Arbaminch, Ethiopia; ^6^ South Ethiopia Region Health Bureau, Jinka, Ethiopia

**Keywords:** newborn cord care, practice, associated factors, chencha town, Southern Ethiopia

## Abstract

**Background:**

Harmful substance-related infections that cause neonatal deaths on the umbilical stump continue to be a major cause of worry, accounting for a large portion of the yearly mortality toll in developing nations such as Ethiopia. In our study region, there is, however, little data regarding these issues. In Chencha town, Southern Ethiopia, mothers who gave birth in the last six months were the subjects of this study.

**Methods:**

A community-based cross-sectional mixed study design was conducted among mothers who gave birth within the past six months, from April to May 2023. Quantitative data was collected through structured interview questionnaires from 312 randomly selected mothers. The collected data was coded, cleaned, and entered into Epi-info version 7.2.5.0, and analyzed using SPSS version 26. Binary logistic regression analysis was used to identify associated factors, and the strength of association was measured by odds ratios with a 95% confidence interval at a *p*-value of <0.05. The audio-recorded qualitative data were transcribed in Amharic language and then translated into English and entered into Open Code software version 3.6.2 for analysis using the thematic content analysis method.

**Results:**

Harmful newborn cord care practice was prevalent among 55.8% (95% CI: 50.1, 61.4) of the mothers. Factors such as husband's educational status (AOR = 3.09, 95% CI: 1.11, 8.67), communication on cord care within the community (AOR = 10.24, 95% CI: 5.44, 19.28), and discussions with health workers regarding cord care (AOR = 7.26, 95% CI: 3.59, 14.64) demonstrated significant associations with harmful newborn cord care practice (*p* < 0.05). In the qualitative analysis, four themes emerged such as substance applied (butter, Vaseline, and ointment). The reasons for application were moisturizing the cord, facilitating its separation, and promoting its healing. The sources of advice on cord care were relatives, neighbors, and HCW.

**Conclusion:**

This study revealed that harmful newborn cord care practice was prevalent among 55.8% [95% CI (50.1, 61.4)] of mothers who gave birth in the last six months. The present study identified husbands' educational status, exposure to cord care messages through interpersonal communication in the community, and mothers who didn't ever engage in discussion with health workers about cord care as significant factors.

## Introduction

Newborn is a term used to describe the first 28 days of a child's life. Globally, the health of newborns has been given priority; however, a significant proportion of newborns suffer various health problems and consequently die (1).

Clean cord care means observing principles of cleanliness throughout labor and delivery and after birth until the separation of the cord stump. To ensure clean cord care in 1994, WHO first identified the “six cleans” that are essential for infection prevention during and after delivery, clean hands, clean birth surface, clean perineum, a clean instrument used to cut the cord, clean cord tie, and a clean cloth for drying (2).

Globally, there have been great strides made in reducing the number of neonatal deaths, declining from 5.1 million in 1990 to 2.4 million in 2019 (3). Despite the progress toward the reduction of under-five mortality globally, newborn deaths have reduced at a slower rate (4). In addition, newborn mortality varies among African nations, from eleven per 1,000 live births in South Africa to 68 per 1,000 live births in Liberia (5, 6). Similarly, in Ethiopia, neonatal mortality declined from 49 deaths per 1,000 live births in 2000 to 28 deaths per 1,000 births in 2017, a reduction of 49% over the past 17 years, which is indicated to be unsatisfactory (7).

The World Health Organization recommends improved newborn care practices at birth to reduce neonatal morbidity and mortality (8). However; cultural practices in people's lives regarding care for newborn cords with topical application of substances on umbilical cord stump persist across cultural contexts all over the globe. It has been speculated that applying something on the cord may indicate that the child may be receiving more care than a child with nothing on the cord; that is, what appears to be dry cord care may indicate neglect (9).

The practice of applying substances on the umbilical cord in Ethiopia was high. Substances are placed on the cord stump to promote healing and separation; and several related traditional beliefs include the prevention of pain, infection, or bleeding, or to keep out evil spirits or cold air. The use of butter, petroleum jelly, and hair lotion for this purpose in Ethiopia contrasts with the use of a wide variety of substances such as cow dung, mustard oil, turmeric, boric powder, ash, and mud in other countries (10).

The use of substances in cord care has long been linked to umbilical cord infection where the devitalized umbilical cord provides an ideal medium for bacterial growth and the patent vessels of the unhealed umbilical cord provide direct communication of microorganisms resulting in local and invasive infections (11, 12)**.** Sources of potential pathogens that colonize the umbilical cord include the mother's birth canal and various local bacterial sources at the site of delivery, most prominently the non-sterile hands of any person assisting with the delivery (13).

Neonatal infection related to harmful umbilical cord care practices accounts for millions of annual neonatal deaths (14). The World Health Organization 2020 report says that among children who die within the first 28 days of birth, almost one-third were due to infections related to inappropriate newborn cord care during the neonatal period (15, 16). Similarly, in Ethiopia neonatal deaths related to infections at the umbilical site from sub-optimal and unhygienic care during and after delivery and application of harmful substances on the umbilical stump continue to be high (9).

Behavior change strategies need to address the local rationales for cord care practices and related issues to effectively bring about improved practices such as optimal cord tying and cutting, and avoiding the application of substances on the cord (14).

The existing evidence on umbilical cord care shows that the practice of good cord care was the predominant topic researched. However, the magnitudes of harmful practice regarding umbilical cord care were poorly understood. Additionally, numerous factors hypothesized to have association lack the facts owing to the limitation in studies investigating harmful umbilical cord care practice and its associated factors.

This necessarily requires investigation on this topic particularly in Ethiopia where the majority of deliveries occur at home, often under sub-optimal and un-hygienic conditions despite the tremendous efforts made in improving the quality and access to health service by implementing a health extension program and community-based newborn care services (15). Therefore, this study aimed to identify the magnitude of harmful newborn cord care practice and its associated factors among mothers, who gave birth in the last six months in Chencha town, Southern Ethiopia.

## Methods

### Study area and period

The study was conducted from April-May, 2023 in Chencha town, SNNP; Ethiopia. Chencha town is located 524 km from the capital city (Addis Ababa), and 270 km from Hawassa (the capital city of the southern nation nationality and people region). The total area of the town is 98 square km and is surrounded by Chencha Zuriya Woreda. Based on the 2022/2023 population projection of SNNPR, Biro of Finance and Economic Department (BOFED), The total population of the town was 94,189 among which 46,058 (49.9%) are males and 48,131 (50.1%) are females. The dominant ethnic group and religious beliefs followed in Chencha town are Gamo and Protestant, respectively. Administratively, Chencha town is divided into 2 kebeles and 5 rural and 3 urban ketenes.

Health services have been provided by public and private health facilities including one primary hospital, one public health center, six private clinics, and two pharmacy/drug stores. The main sources of income are waving and agriculture particularly apple production is the main cash crop. Regarding the educational sector, there is one TVT college, one preparatory and 3 high schools, and 12 elementary and junior schools. Utilities such as clean pipe water supply, hydroelectric power, telecommunication, and postal services were also available in the town.

### Study design

A community-based cross-sectional and phenomenological study design that employed a quantitative followed by qualitative method of data collection was conducted.

## Source population and study population

### Source population

The source population of this study was all mothers with less than six months baby before the study period in Chencha town.

### Study population

The study population was all mothers with less than six months baby during the study period in Chencha town.

### Study units

Randomly selected mothers with less than six months of baby.

### Eligibility criteria

#### Inclusion criteria

The inclusion criteria were the provision of consent to participate in the study, the period of having a baby less than six months, and residents for at least 6 months in the study area were included in this study.

#### Exclusion criteria

The exclusion criteria were unable to respond due to illness.

### Sample size determination

The sample size for the first objective (prevalence of harmful newborn cord care practice) is determined by using a single population proportion formula with the following assumption: the two-sided confidence level of 95% with the corresponding Z score of 1.96, the margin of error of 4%, the prevalence of harmful newborn cord care practice (13.7%) from a study investigating individual and community-level factors associated with the application of cow dung and oil on the umbilical cord stump in Ethiopia (17).

The sample size for the first objective (prevalence of harmful newborn cord care practice) was 284.

$$N=\frac{{\left(z_{\frac{\alpha }{2}}\right)}^{2}*\;p(1-p)}{d^{2}}=\frac{{\left(1.96\right)}^{2}\;*\;0.137(1-0.137)}{0.04^{2}}=284$$

The sample size for the second objective was determined with Epi info version 7.2.5.0 statistical packages by assuming the ratio of unexposed to exposed in the sample, percent of exposed with outcome, percent of unexposed with Outcome at CI, and power of 95% and 80%, respectively.

The factors considered in sample size determination were level of education (no formal education), Occupation (housewife), Place of delivery (home) from a study conducted in MizanTepi Ethiopia in 2021 (18) and also *ANC* (no) and Parity (multipara) from a study done in Tabora region (19) Table 1.

**Table 1:** Sample size determination for factors associated with harmful newborn cord care practice.

Variables	Two-sided confidence level (%)	Power	The ratio of unexposed to exposed in the sample	% of exposed with outcome	% of unexposed with outcome	Sample size
Level of education (no formal education)	95%	80%	1	63	30	82
Occupation (housewife)	95%	80%	1	44.78	26.47	236
Place of delivery(home)	95%	80%	1	81.82	42	56
Parity(multipara)	95%	80%	1	68.2	50.6	266
*ANC* *(no)*	*95%*	*80%*	*1*	*93*.*5*	*53.53*	*46*

The final sample size from the first objective (prevalence of harmful newborn cord care practice) which yielded the largest sample size estimate was 312 after adding a 10% non-response rate (284 + 28). Sample sizes for the qualitative study were determined based on the number of interviews expected to capture the variety of respondent practices and perspectives and to reach saturation point (i.e., sampling until no new information emerged).

### Sampling method and procedure

The sampling method that was employed in this study followed a simple random sampling technique. First, a sampling frame of households with mothers who gave live birth in the last six months was prepared by Reviewing the Health Post Family folder and records from all kebeles (2) in Chencha town. Then, study participants were selected using a computer-generated random number method from households in the sampling frame. Finally, during fieldwork data collectors identified selected study participants using identifiers such as Kebele, ketena, mender, and household number from the sampling frame of the household. In the case of no mother of the study population identified in the selected household, the next household has been visited. Whenever more than one eligible respondent was found in the same selected household, only one respondent was chosen by lottery method.

Study participants for the qualitative study were selected purposively. To represent varying perspectives on cord care and predominant culture and traditional beliefs within a community, data collectors selected mothers who gave birth at health facilities and homes as well as urban and rural residents.

### Data collection tools and procedure

Quantitative data was collected using a structured questionnaire, which is adapted from various literature (13, 15–17). The questionnaire written in English was translated into the local language (Amharic) and back to English to keep consistency. A pre-test of 10% of mothers with similar characteristics at Kogota Woreda was conducted before the actual data collection for internal consistency. The Cronbach's alpha was determined and it was 0.83 which indicated that the internal consistency of the questionnaire is acceptable. The questionnaire is designed to gather information regarding the socio-demographic characteristics, obstetric and health service utilization, and knowledge of cord care.

The qualitative data was audio-recorded during semi-structured in-depth interviews with open-ended questions which were adapted from various literatures. The interview guide included questions on local perceptions and concerns -which mirror values, customs, and beliefs- regarding desired outcomes with respect to the cord and their role in harmful newborn cord care practices.

The data was collected by 4 diploma nurses who are fluent speakers of the local languages through face-to-face interviews under close supervision and facilitation by one health officer and one BSc nurse as a supervisor and the principal investigator as necessary.

### Descriptions of variables

#### Dependent variables

Harmful newborn cord care practice.

#### Independent variables

It includes socio-demographic and economic factors such as maternal age, maternal educational status, maternal occupation, wealth status, place of residence, newborn's gender, marital status, husband's education, and husband's occupation; obstetric and health service utilization-related factors like parity, ANC follow-up, place of delivery, and postnatal follow-up. It also involves mother's knowledge and cultural and belief factors such as level of knowledge of cord care, health worker discussion on cord care, interpersonal communication on cord care in the community, and culture/traditions, beliefs.

### Operational definitions

Harmful cord care practice is defined in this study as the application of any harmful substances on the umbilical cord stump other than 4% chlorhexidine (20). Those mothers who applied any substance on the umbilical cord stump other than 4% chlorhexidine will be categorized as “Yes” and coded as “1”. Those mothers who didn't apply any substance on the umbilical cord stump other than 4% chlorhexidine were categorized as “no” and was coded as “o”.

Knowledge of mothers about newborn cord practices: Knowledge regarding umbilical cord care was assessed using 6 items in multiple choice questions and measured on a ratio scale. One point was awarded for a correct response and 0 for any incorrect response. The total score was 6 points, and mothers who scored more than 50% of the total score were considered as having good knowledge, and those who scored below 50% were considered as having poor knowledge (21).

### Household wealth index

Asset information was collected based on the kinds of consumer goods that households owned, factor scores were produced using PCA, and the composite scores were further separated into five quantiles. The first 20 percent quantile was determined to be the poorest, and the last 20 percent quantile to be the richest.

### Data quality control

To ensure data quality, the questionnaire was carefully designed and translated. The training was given for two days about the objective, the relevance of the study, confidentiality of information, respondent's rights, informed consent, and techniques of interview. Also, a practical demonstration of the interview was carried out in the classroom.

Moreover, close supervision was undertaken by supervisors and the principal investigator during the data collection process. Concomitantly, the collected data was checked for completeness and clarity by the principal investigator and supervisors daily to ensure quality data.

### Data processing and analysis

Data was entered into Epi-info software version 7.2.5.0 and clean-up and cross-checking was made. Univariate data analysis was conducted to examine the distribution of studied variables and summarize using summary statistics and graphical techniques. Continuous variables were reported using summary statistics such as mean, median, standard deviation, and interquartile range whereas frequencies and percentages were used as summary statistics for categorical variables. In addition, a graphical technique such as aspire charts was used to summarize the distribution of studied variables.

Then, bivariate analysis using binary logistic regression was carried out to describe the relationship between studied variables and harmful newborn cord care practice. Variables with *p*-value ≤ 0.25 in the bivariate analysis will be selected as candidate variables for multiple logistic regression analysis to control the effect of confounders.

A multi-co linearity test was performed to check the correlation between independent variables using co-linearity statistics (variance inflation factors >5 was considered suggestive of multi-co linearity). The model fitness was checked using Hosmer and Lemeshow statistics and a *p*-value > 0.05 was considered a good model fit. A variable with a *p*-value of less than 0.05 in multiple logistic regression analysis was considered to have a significant association with the outcome and adjusted odds ratios with their 95% confidence intervals were reported for those explanatory variables.

The analyses of the qualitative data were made using the thematic content analysis method. First, audio-recorded interviews were transcribed in Amharic language. Then, the transcripts were translated into English and entered into Open Code software version 3.6.2 for analysis. The list of themes to be used for coding was developed on the basis of the questions in the interview guides and on expected responses based on previous studies. This list was supplemented with additional themes during the coding process. The data within each theme was reviewed, synthesized, and written up. Participant statements that are especially expressive, informative, and representative were selected and presented as quotes.

## Result

### Socio-demographic characteristics of study participants

A total of 312 mothers who gave birth in the last six months participated in the interviews, resulting in a response rate of 100%. Table 2 presents the percentage distribution of mothers and their index children based on background characteristics. The average age of mothers with index children was 29 years (SD ± 6), with 135 of them (43.3%) aged 30 years or older.

**Table 2:** Socio-demographic characteristics of study participants in Chencha town, southern Ethiopia, 2023 (*n* = 312).

Characteristics		Frequency	Percentage
Maternal age (years)	24 or less	77	24.7
	25–29	100	32.1
	30 or above	135	43.3
Religion	Protestant	170	54.5
	Orthodox	142	45.5
Maternal educational status	No formal education	69	22.1
	Primary	63	20.2
	Secondary	109	34.9
	College and above	71	22.8
Maternal occupational status	Housewife	126	40.4
	Merchant	24	7.7
	Government employee	46	14.7
	Self-Employee	58	18.6
	Student	30	9.6
	Farmer	28	9.0
Place of residence	Urban	187	59.9
	Rural	125	40.1
Marital status	Never married	19	6.1
	Currently married	293	93.9
Husband education status	No formal schooling	41	13.7
	Primary school completed	48	16.1
	Secondary school completed	120	40.1
	College and above	90	30.1
Husband occupation	Merchant	44	14.7
	Government employee	69	23.1
	Self-Employee	110	36.8
	Farmer or weaver	76	25.4
Newborn sex	Male	145	46.5
	Female	167	53.5
Wealth status	1st quintile	60	19.3
	2nd quintile	64	20.6
	3rd quintile	65	20.9
	4th quintile	60	19.3
	5th quintile	62	19.9

Regarding religious affiliation, more than half of the study participants (170, 54.5%) were Protestants, while 142 (45.5%) identified as Orthodox. The distribution by place of residence and marital status reveals that 187 (59.9%) participants reside in urban areas, and 293 (93.9%) of the mothers were currently married. In terms of education, more than one-third of the mothers (34.9%) and their partners (40.1%) completed secondary school. Regarding occupation, 126 (40.4%) mothers and 110 (36.8%) fathers of the index children were housewives and self-employed, respectively.

Furthermore, the median age of the index children was 4 months, with the largest proportion (167, 53.5%) being female. Additionally, when considering the wealth index, a relatively higher proportion of mothers and their index children (124, 39.9%) were categorized in the poorest quintile Table 2.

### Prevalence of harmful newborn cord care practice

**Figure 1:**
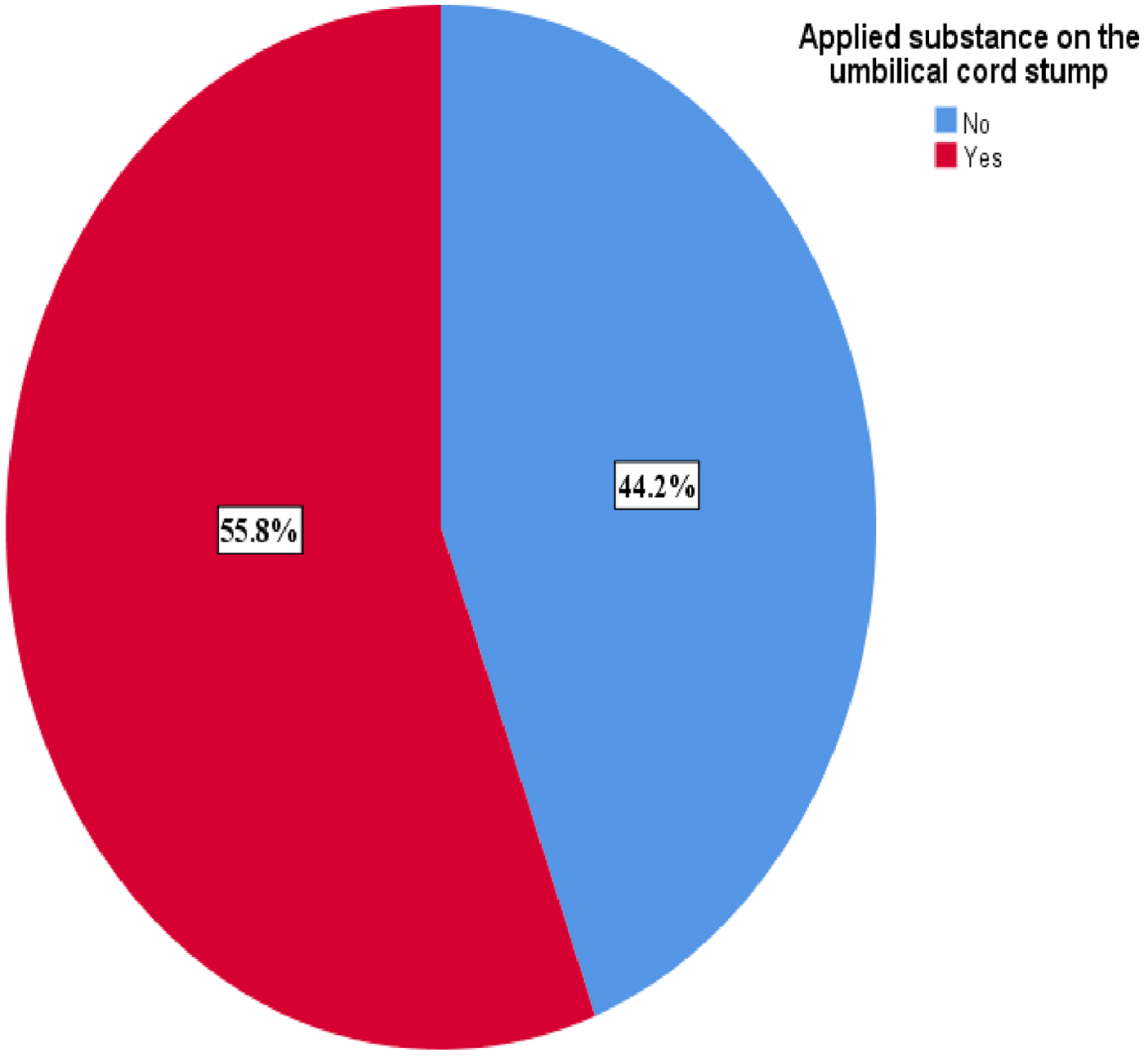
Prevalence of newborn cord care malpractice among mothers, who gave birth in the last six months in Chencha town, Southern Ethiopia, 2024 (*n* = 312).

The finding shows that harmful newborn cord care practice was prevalent among 55.8% [95% CI (50.1, 61.4)] of mothers, who gave birth in the last six months in the study area Figure 1.

### Factors associated with harmful new-born cord care practice

The finding of this study showed an association between harmful newborn cord care practice among mothers, who gave birth in the last six months and social and maternal age, educational status, place of residence, husband educational status, and wealth status in bivariate logistic regression analysis at a *p*-value of 0.25. Moreover, exposure to cord care messages on mass media, antenatal care, postnatal care utilization, mother's knowledge, and interpersonal communication on cord care in the community and health workers discussed cord care showed association with harmful newborn cord care practice among mothers, who gave birth in the last six months in bivariate logistic regression analysis at a *p*-value of 0.25**.**

The multivariable logistic regression analysis showed an association between harmful newborn cord care practice among mothers, who gave birth in the last six months and their husband's educational status, exposure to cord care messages through interpersonal communication in the community, and ever engagement in discussions with health workers about cord care in at a *p*-value of 0.05.

The likelihood of harmful newborn cord care practice was 3.09 (1.11, 8.67) times higher for mothers whose partners had no formal schooling than those with the educational status of college and above. The odds of harmful newborn cord care practice among mothers whose major sources of information on cord care were family, relatives, neighbors, or the community were 10.24 [95% CI (5.44, 19.28)] times higher than their counterparts. Moreover, mothers who didn't ever engage in discussion with health workers about cord care were 7.26 [95% CI (3.59, 14.64)] times more likely to have harmful newborn cord care practice than those who ever engaged in discussion with health workers about cord care. Please refer to Table 3.

**Table 3:** Factors associated with harmful newborn cord care practice in chencha town, southern Ethiopia, 2023 (*n* = 312).

Variables	Harmful practice		COR (95% CI)	AOR (95% CI)	*p*-value
	Yes	no			
Maternal age (Reference group 25–29 years)	46 (26.4%)	54 (39.1%)	1	1	1
24 or less	47 (27.0%)	30 (21.7%)	1.84 (1.01, 3.36)	1.76 (.74, 4.15)	.200
30 or above	81 (46.6%)	54 (39.1%)	1.76 (1.04, 2.97)	1.09 (.53, 2.27)	.812
Maternal educational status (Reference group college and above)	33 (19.0%)	38 (27.5%)	1	1	1
No formal education	53 (30.5%)	16 (11.6%)	3.81 (1.84, 7.90)	1.84 (.57, 5.90)	.306
Primary	44 (25.3%)	19 (13.8%)	2.67 (1.31, 5.43)	.95 (.33, 2.72)	.931
Secondary	44 (25.3%)	65 (47.1%)	.78 (.43, 1.42)	.65 (.28, 1.50)	.313
Place of residence (Rural)			1.41 (.89, 2.23)	.85 (.35, 2.06)	.719
Husband's education status (Reference group college and above)	42 (25.6%)	48 (35.6%)	1	1	
No formal education	32 (19.5%)	9 (6.7%)	4.06 (1.74, 9.48)	3.09 (1.11, 8.67)	.031*
Primary school completed	24 (14.6%)	24 (17.8%)	1.14 (.57, 2.30)	.62 (.25, 1.49)	.285
Secondary school completed	66 (40.2%)	54 (40.0%)	1.39 (.81, 2.42)	1.41 (.69, 2.87)	.347
Wealth status (Reference group 5th quintile)	24 (13.9%)	38 (27.5%)	1	1	
1st quintile	40 (23.1%)	20 (14.5%)	3.17 (1.51, 6.64)	2.25 (.74, 6.84)	.152
2nd quintile	35 (20.2%)	29 (21.0%)	1.91 (.94, 3.88)	1.54 (.54, 4.37)	.419
3rd quintile	40 (23.1%)	25 (18.1%)	2.53 (1.24, 5.18)	2.10 (.76, 5.79)	.150
4th quintile	34 (19.7%)	26 (18.8%)	2.07 (1.01, 4.26)	2.45 (.93, 6.45)	.071
Antenatal care (Reference group 4 ANC visit)	52 (29.9%)	76 (55.1%)			
No ANC visit	38 (21.8%)	8 (5.8%)	6.94 (2.99, 6.08)	1.77 (.55, 5.77)	.340
1–3 ANC visit	84 (48.3%)	54 (39.1%)	2.27 (1.39, 3.72)	1.58 (.78, 3.20)	.200
Place of current delivery (Reference group Government Hospital)	92 (52.9%)	107 (77.5%)	1	1	
Home	56 (32.2%)	10 (7.2%)	6.51 (3.14, 3.49)	1.78 (.55, 5.78)	.335
Health center	26 (14.9%)	21 (15.2%)	1.44 (.76, 2.73)	1.03 (.40, 2.66)	.947
Postnatal care (Reference group 2 or more PNC visits)	41 (23.6%)	34 (24.6%)	1	1	
No PNC visit	47 (27.0%)	12 (8.7%)	3.25 (1.49, 7.09)	.80 (.26, 2.47)	.705
1 PNC visit	86 (49.4)	92 (66.7)	.77 (.45, 1.33)	.66 (.30, 1.42)	.289
Mothers’ knowledge of cord care (Poor)	94 (54.0)	53 (38.4)	1.88 (1.19, 2.97)	.89 (.44, 1.84)	.766
Interpersonal communication on cord care in the community (Yes)	48 (27.6)	110 (79.7)	10.31 (6.06, 17.55)	10.24 (5.44, 19.28)	.001**
The health worker discussed cord care (No)	93 (53.4)	15 (10.9)	9.41 (5.09,17.38)	7.26 (3.59, 4.64)	.001**

Where *is a *p*-value less than or equal to 0.05 and greater than 0.01, **is a *p*-value less than or equal to 0.01, and 1 is a reference group.

### Qualitative findings

In this study four themes emerged with some properties such as substance applied, reason for application or not application, sources of advice on cord care, and cord-related risk and infection. Each theme is presented in detail with appropriate descriptions and quotes cited in the text to support the categories.

### Substance applied

Among mothers who applied substance on the umbilical cord stump, the majority of them used butter followed by Vaseline and ointment. Preference regarding the type of substance to use was found to be related to the effect on moistening and smoothing the umbilical cord stump. The substances applied were also believed to moisturize the cord to prevent sores and promote healing. This reason was mentioned mostly by a 35-year-old mother in Chencha town elaborated: “*We apply butter on the edge of the cord to speed up cord separation and in the space that opens up as the cord drops off so that it will be smooth and moisten”*.

Also, some mothers reported the application of chlorohexidine but not any other substance as recommended by health workers. For instance, a 27-year-old mother in Chencha town said that

“I gave birth at Chencha hospital and the health worker counseled me to apply chlorohexidine two times daily until the separation of the cord stump on the edge of the cord to prevent infection, promote healing, and speed up separation. I applied two times a day for two days”.

### Reason for application or not application

Among the reasons cited by study participants, moistening and softening the umbilical cord stump is the main reason mothers apply substances on the umbilical cord stump. For instance, a 36-year-old mother mentioned “*We applied butter on the cord reason to softening, promoting healing and speed up separation if we do not apply the cord may be ulcerate, bleed and cracking*”.

The majority of mothers who didn't apply the substance on the umbilical cord stump stated that they were informed on how to care for the cord care. For instance, a 32-year-old mother in Chencha town stated “*I didn't apply anything on the cord because it will fall off even if it becomes dry.”*

### Sources of advice on cord care

Availability of appropriate information on cord care is vital to its practices, in this study, more than half of the mothers were aware of the details of standard cord care. The main source of information reported by most study participants was interpersonal communication. For example, *a 35-year-old mother in Chencha town stated* “*My mother and neighbor advised me to apply butter on the cord that can moisten, promote healing, and speed up healing”*.

Some mothers cited health workers as their source of information on how to care for cords.

For instance, a *30-year-old mother in Chencha Town stated* “*Health workers advised me only to bathe the cord, to avoid any substance on the cord at home or to go to a health facility in case of a sore, bleeding”*.

### Cord-related risk and infection

The level of understanding of risks and infections affecting the cord and efforts to prevent and respond to them is low in the study area. Many mothers state that they are unaware of any risks or problems affecting the cord.

Sores bleeding discharge, and failure to heal properly are the most reported cord-related risks, and infection and application of harmful substances have been linked with efforts to prevent and respond to this condition in this study. For example, a 30-year-old mother in Chencha said “*The cord may develop sores, bleeding. If we do not bathe and massage the cord with butter until it is separated, the edge of the cord may crack and bleed”*.

Similarly, a 28-year-old mother in Chencha Town said, “*The cord may develop sores, bleeding. If we do not apply butter on the cord until separation the cord may ulcer, discharge, and bleeding.”*

## Discussion

The findings of this study indicate that there is a high prevalence of harmful newborn cord care practice among mothers who gave birth in the study area within the last six months, with 55.8% (95% CI: 50.1, 61.4) of mothers engaging in such practices. A majority of mothers reported using butter, followed by Vaseline and ointment to treat the umbilical cord stump. The choice of substance used was often influenced by the perceived effects of moistening and smoothing the stump.

These findings align with previous studies conducted in Gulo Mekeda, Ethiopia, where approximately 60% of mothers reported applying butter or oil on the cord stump (21). Similarly, a study conducted in Mizan Tapi, Ethiopia in 2021 reported that more than half (54.7%) of the participants applied unnecessary substances to the cord (18). This similarity in findings suggests a common practice across different regions, which may be influenced by socio-economic factors within the respective study populations.

It is important to consider the socio-economic context when designing interventions to address harmful newborn cord care practice. By understanding the factors influencing these practices, tailored behavior-change strategies can be developed to promote safe and hygienic cord care practices among mothers, ultimately reducing the risk of neonatal infections and mortality.

In contrast to this, the finding is higher than in a study conducted in Ethiopia 13.70% of mothers applied potentially harmful substances (22). And study conducted in East Gojjam shows that 5.4% of the respondents reported that they had substance application on the umbilical stamp (23). This might be due to the difference in the study period and setting and the way they defined harmful practice.

Husband's educational status was found to have a significant association with harmful cord care practices. In particular, the likelihood of harmful newborn cord care practice was higher for mothers whose partners had no formal schooling than those with the educational status of college and above. Similarly, a study conducted in Rwanda investigated husband's educational status effect on harmful cord care practice but failed to establish an association (24). The findings of this study could be explained by the fact that the husband's education improves familial access to new information and interaction with healthcare providers and brings effective negotiation within familial power structures about beneficial child care.

According to this study, exposure to cord care messages through interpersonal communication in the community and harmful newborn cord care practice among mothers, who gave birth in the last six months. In particular, Mothers who relied on family, relatives, neighbors, or the community as their primary sources of information on newborn cord care were more likely to engage in harmful practices compared to others. Although previous studies failed to establish this association, this finding is supported by qualitative findings of the current study as the majority of study participants cited relatives and neighbors as their source for information about how to care for the umbilical cord stump. For instance, a 35-year-old mother in Chencha town stated “*My mother and neighbor advised me to apply butter on the cord that can moisten, promote healing, and speed up healing*.”

The finding of this study showed**,** that mothers who didn't ever engage in discussion with a health worker about cord care were 7.26 times more likely to have harmful newborn cord care practice than those who ever engaged in discussion with a health worker about cord care. This finding is in line with qualitative findings of the current study where the majority of mothers state that they were unaware of risks and infections affecting the cord and efforts to prevent and respond to them. The reason for this could be the fact that these mothers who didn't ever engage in discussion with health workers about cord care are more likely to be influenced by social customs or were unaware of the health risks involved related to the application of potentially harmful substances to the stump of the umbilical cord.

## Conclusion and recommendation

This study highlights the prevalence of harmful newborn cord care practice among 55.8% of mothers who gave birth within the last six months in the study area. The use of substances such as butter, Vaseline, and ointment for cord care was common, with preference influenced by the perceived effects on moistening and smoothing the umbilical cord stump. Additionally, factors such as the husband's educational status, exposure to cord care messages through interpersonal communication in the community, and lack of discussion with healthcare workers regarding cord care were identified as significant factors contributing to harmful newborn cord care practice. Given the high prevalence of harmful newborn cord practice, special attention needs to be given to cord care in healthcare settings. This includes reinforcing standardized protocols, conducting regular audits, and implementing quality improvement initiatives to ensure adherence to recommended cord care practices. Hospital administrative staff and health bureaus need to encourage healthcare providers working in the Neonatal Intensive Care Unit (NICU) to closely monitor and promote proper delivery care and newborn care practices, including cord care. Regular training and refresher courses on cord care guidelines should be provided to healthcare professionals.

## Data Availability

The raw data supporting the conclusions of this article will be made available by the authors, without undue reservation.
