# Supplementary material for: Harmful newborn cord care practices and associated factors among mothers who gave birth in the last six months in Chencha town, Southern Ethiopia: a mixed-methods study
Source: Front Pediatr. 2025 Jan 28;12:1492222. doi: 10.3389/fped.2024.1492222 (PMC11810915; doi:10.3389/fped.2024.1492222)
Supplement: Supplementary file 1 [file Datasheet1.zip › supplementary/amharric version questinaire.DOCX]

## ANNEX II: ለጥናቱተሳታፊዎችመረጃለመስጠትየተዘጋጄቅጽ

ለቃለመጠየቅየግለሰብምላሽሰጪዎችየጥናቱንመረጃመስጫወረቀትእናበመረጃላይየተመሰረተየስምምነትመፈራረሚያቅጽ

የእኔስም ----------------------------------- ነው፡፡በአርባምንጭ ዩኒቨርሲቲ የማስተርስ (MPH) ተማሪየሆነውምስጋናሠይፉከአርባምንጭዩኒቨርሲቲ፣ጋርበጨንቻከተማአስተዳደርየጨቅላህጻናትትክክለኛያልሆነየዕትብትእንክብካቤእናአጋላጭችግሮችንበእናቶችለማጥናትለማሰስእያካሄዴላለውጥናትመረጃሰብሳቢሆኜእየሰራሁነው። ስለሆነምስለጥናቱማብራሪያእንድሰጥህትኩረትእንድትሰጪኝበትህትናእጠይቃለሁ።

**የጥናት ርዕስ፡** በጨንቻከተማአስተዳደርየጨቅላህጻናትትክክለኛያልሆነየዕትብትእንክብካቤእናአጋላጭችግሮችንበእናቶችማጥናትናማሰስ፡፡ስሆንጥናቱየሚካሄደውበደቡብኢትዮጵያ፤በደቡብጋሞዞን፣በጨንቻከተማአስተዳደርውስጥነው።

**የጥናቱአላማ፡**የዚህጥናትዋናአላማየስነተዋልዶየህብረተሰብጤናየማስተርስድግሪ (MPH)/ተመራቂተማሪውየመመረቂያጥናታዊጽሁፍማቅረብየማስተርስድግሪትምህርቱንለማጠናቀቅእንደከፊልመስፈርትሆኖስለምያዝነው። በተጨማሪምየጥናቱውጤትለአርባምንጭዩኒቨርሲቲ፣ለቀጣይበጨቅላህጻናትየሚደርስትክክለኛያልሆነየዕትብትእንክብካቤእናአጋላጭችግሮችንለመከላከልስትራቴጂነድፎተግባራዊእንቅስቃሴለማድረግእንደማስረጃእናግብአትነትያገለግላል።

**የጥናቱ ግብእናእነማንይካተታሉ፡- የጨቅላህፃናትየሚደርስትክክለኛያልሆነየዕትብትእንክብካቤእናአጋላጭችግሮችንማረጋገጥ**፡፡ በዚህጥናትጨንቻከተማየጨቅላህፃናትየሚደርስትክክለኛያልሆነየዕትብትእንክብካቤእናአጋላጭችግሮችንበመለየትየጨቅላህፃናትጤናንማረጋገጥማጥናትእንፈልጋለን። በሚገኘውየጥናትውጤትምለወደፊትስልቶችንለመንደፍበጣምአስፈላጊነው።

**የጥናቱሂደትእናየቆይታጊዜ፡** ብቁእናየሰለጠኑመረጃሰብሳቢዎችለጥናቱአጋዥየሆነበቂመረጃመሰብሰብእንድቻልለዝሁጥናትየተዘጋጄየቃለመጠይቅመምሪያበመጠቀምአስፈላጊውንመረጃከተጠያቂዎችይሰበስባሉ። ሰለቅድሜትብብሮ፣በአብሮኑሮነት፣ማህበራዊሁኔታዎች፣እናየኑሮሁኔታጥያቄዎችይጠየቃሉ። የሚደረገውቃለ-መጠይቅፍት-ለፍትጥያቄይጠየቃሉእናምበወረቀትይገለበጥናይተረጎማል፡፡የዝህቃለ-መጠይቅይዘትበተመራማሪውቡድንይተነተናልእንጂለሌሎችሰዎችአይጋራም። የቃለ-መጠይቁቆይታጊዜበአማካይ ከ40-45 ደቂቃዎችሲሆንቢበዛአራትክፍሎችይኖሩታል።

**ጉዳትእናአለመመቸት፡** ከተለመደውበውይይትወቅትልኖሩከምችሉሁኔታዎችበተለየከዚህጥናትጋርልመጣየሚችልምንምአይነትጉዳትይኖራልብለንአንጠብቅም።

**የጥናቱጥቅሞች፡** የእርስዎተሳትፎበጨንቻከተማየጨቅላህፃናትየሚደርስትክክለኛያልሆነየዕትብትእንክብካቤእናአጋላጭችግሮችለመረዳትይረዳናል፡፡ ይህመረጃየጨቅላህፃናትየሚደርስትክክለኛያልሆነየዕትብትእንክብካቤእናአጋላጭችግሮችለደረሰባቸውህጻናትቀጥተኛምላሽባይሰጥምበጨቅላህፃናትየሚደርስትክክለኛያልሆነየዕትብትእንክብካቤእናአጋላጭችግሮችላይችግሩንለመፍታትመረጃይሰጠናል፡፡

**ሚስጥራዊነት**፡ ከእርስዎየተገኘመረጃሚስጥራዊይሆናል። በተለየሁኔታተለይቶየሚታወቅመረጃአይኖርም፡፡ የጥናቱግኝቶችለጥናቱአጠቃላይለእናቶችህጻናትይሆናል፡፡ እናምየግለሰቦችንየተለየነገርአያንፀባርቅም። የመረጃመሰብሰብያቅጾችበሙሉስሞችንእናሌሎችየግልመረጃዎችንከማሳየትለማስቀረትበኮድብቻይመዘገባሉ። ተሳታፊዎችንከጥናቱጋርሊያገናኙየሚችሉየቃልወይምየጽሁፍዘገባዎች/ሪፖርቶችማጣቀሻአይደረግም።

**ጥናቱንያለመቀበልወይምከጥናቱጥሎየመውጣትመብቶች፡-**በዚህጥናትለመሳተፍመስማማትሙሉበሙሉበፈቃደኝነትላይየተመሰረተነው። ለዚህጥናትፍቃድየመስጠትመብትአላችሁ ።ጥናቱንለመሳተፍከወሰንሽ/ከወሰንክበኋላምከጥናቱጋርየተያያዘነገርየማይመችእንደሆነካሰቡበማንኛውምጊዜጥናቱንየማቋረጥመብትምአለሽ፡፡

**ተቋማዊእናስነ-ምግባርኮሚቴዎች፡-** ይህጥናትበሚከተሉትየግምገማቦርዶችጸድቋል፡- የአርባምንጭዩኒቨርሲቲህክምናናጤናሳይንስኮሌጅየተቋማትገምጋሚ ​​ቦርድ፣የስነ-ምግባርኮሚቴውአጽድቋል፡፡

**ጥያቄካለማግኘትየሚችሉትሰውአድራሻ**፡- በኋላላይበዚህጥናትውስጥስለመሳተፎማንኛውምአይነትጥያቄካሎት፣ኃላፊነትየሚሰማቸውንማነጋገርይችላሉ። ማንኛውምአይነትጥያቄአለ?

ዋናመርማሪ፡- አቶምስጋናሠይፉአርባምንጭዩኒቨርሲቲየህክምናናጤናሳይንስኮሌጅየህብረተሰብጤናትምህርትክፍልተማሪ፡- በስልክቁጥር 0916854615 ኢሜል፡ misganaseifu34@gmail.com

አቶሱልጣንሁሴንአርባምንጭዩኒቨርሲቲየህክምናናጤናሳይንስኮሌጅየህብረተሰብጤናትምህርትክፍልመምህርየጥናቱዋናአማካሪበስልክቁጥር 0933539505 ኢሜል፡ sultanhussn@gmail.com

አቶመቅድምካሳአርባምንጭዩኒቨርሲቲየሕክምናናጤናሳይንስኮሌጅ; የህብረተሰብጤናትምህርትክፍልመምህርየጥናቱረዳትአማካሪበስልክ፡ 0911952618 ኢሜል mekidimka@gmail.com

ስምምነት ፡ በዘህፎርምጥናቱንበማስመልከትየቀረበውንሁሉንመረጃአንብቤበጥናቱለመሳተፍተስማምቻለሁ፡፡

ስም…………………………………………………………..ፍረማ…………………………ቀን……………………………………

በጥናቱለመሳተፍካልተስማሙበፎርሙላይመፈረምአይጠበቅቦትም

መረጃሰብሳቢፍረማ………………………………….........ቀን…………………………

ANNEX V: የአማረኛመጠይቅቅጽ

| መግለጫ | | | መለያ | |
| --- | --- | --- | --- | --- |
| ክልል | | |  | |
| ዞን | | |  | |
| ወረዳ | | |  | |
| ቀበሌ | | |  | |
| የሱፐርቫይዘርስምናፊርማ | | |  | |
| የመረጃሰብሳቢውስምናፊርማ | | |  | |
| የተሳታፊመለያኮድ | | |  | |
| ቀን | | | ______/______/2023 | |
| **1.ማህበራዊ፣ስነ- ህዝባዊእናኢኮኖሚያዊጥያቄዎች** | | | | **ወደ**  **ሚቀጥለው**  **ተሻገር** |
| ተ.ቁጥር | ጥያቄዎች | የመልስአማራጮች | |  |
| 101 | የተሳታፊእድሜ | [______________] | |  |
| 103 | ሐይማኖት | 1. ኦርቶዶክስ [___] 2. ሙስሊም [___] 3. ፕሮቴስታንት [___] 4. ካቶሊክ[___] | |  |
| 102 | የትምህርትደረጃ | 1.መፃፍማንበብየማይችል/የማትችል[___]  2.መፃፍማንበብየሚችል/የምትችል [___]  3.1ኛደረጃ/1-8ክፍል/ [___]  4. 2ኛደረጃ/9- 12 / [___]  5.ዲፕሎማናከዚያበላይ [___] | |  |
| 104 | የስራሁኔታ | 1. የቤትእመቤት  2. ነጋዴ  3. የመንግስትሰራተኛ  4. የግልስራ  5. ተማሪ  6. ሌላካለይገለጽ--------------------- | |  |
| 105 | የሚኖሩበትቦታ | ከተማ.........................................1  ገጠር...........................................2 | |  |
| 106 | የትዳርሁኔታ | 1. ያላገባ  2. ያገባ  3. አግብቶየፈታች  4. ባልየሞተባት | |  |
| 107 | የባልየተ/ትደረጃ | 1. መደበኛ ት/ት ያልተከታተለ  2. የመጀመሪያደረጃ  3. ሁለተኛደረጃ  4. ኮሌጅእናከዚያበላይ | |  |
| 108 | የባልየስራሁኔታ | 1. ነጋዴ  2. የመንግስትሰራተኛ  3. የግልስራ  4. ተማሪ  5. ሌላካለይገለጽ--------------------- | |  |
| 109 | የልጅሽፆታምንድነው | ወንድ-------------------------------1  ሴት----------------------------------2 | |  |
| 110 | የልጅሽዕድሜስንትነው | በቀንይገለፅ---------------------------- | |  |

**2. የቤተሰብየሃብትምጣኔሁኔታእናተያያዥጥያቄዎች**

| 201 | በቤትዎውስጥስንትክፍሎችለመኛታያገለግላሉ? |  | [___________] | |
| --- | --- | --- | --- | --- |
| 202 | ለቤተሰብህአባልየመጠጥውኃዋነኛምንጭምንድንነው? | 1 | የህዝብቧንቧውሃ | |
|  |  | 2 | የቧንቧውሃወደጎረቤትየተጠለፈ | |
|  |  | 3 | የቧንቧውሃወደግቢየተጠለፈ | |
|  |  | 4 | የቧንቧውሃወደመኖሪያቤትየተጠለፈ | |
|  |  | 5 | የታሸገውሃ | |
| 203 | የተለየወጥ/ኩሻና/ ቤትአለህ? | 1 | አዎ | |
|  |  | 2 | አይ | |
| 204 | ይህቤትየሚጠቀመውበምንዓይነትየመጸዳጃቤትውስጥነው? | 1 | የውሃፍሳሽ ያለውመጸዳጃቤት | |
|  |  | 2 | የተነባበረየጉድጓድመጸዳጃቤት | |
|  |  | 3 | መጸዳጃቤትየለም | |
|  |  | 4 | ሌላ............. | |
| 205 | ቤተሰቦችህምግብለማብሰልበአብዛኛውየሚጠቀሙትምንዓይነትነዳጅነው? | 1 | ኤሌክትሪክ | |
|  |  | 2 | እንጨት | |
|  |  | 3 | ከሰል | |
|  |  | 4 | ባዮጋስ | |
|  |  | 5 | ተፈጥሯዊ | |
|  |  | 6 | ሌላ | |
| 206 | በቤትዎውስጥ |  | አዎ | አይ |
|  | ኤሌክትሪክአለ? |  | 1 | 0 |
|  | ሬድዮአለ? |  | 1 | 0 |
|  | ቴሌቭዥንአለ? |  | 1 | 0 |
|  | የቤትስልክአለ? |  | 1 | 0 |
|  | ፍሪጅአለ? |  | 1 | 0 |
|  | ጠረጴዛአለ? |  | 1 | 0 |
|  | ሶፋ/ ወንበርአለ? |  | 1 | 0 |
|  | አልጋእናከጥጥ/እስፓንጅ/ እስፕርንግየተሰራፍራሻአለ? |  | 1 | 0 |
|  | የኤሌክትሪክምጠድአለ? |  | 1 | 0 |
| 207 | ከቤተሰብዎአባልውስጥየሚከተሉትንቁሶችያለውአለ? |  | አዎ | አይ |
|  | የእጅሰዓት? |  | 1 | 0 |
|  | ሞባይል? |  | 1 | 0 |
|  | ሳይክል? |  | 1 | 0 |
|  | የሞተርሳይክል? |  | 1 | 0 |
|  | ባጃጅ? |  | 1 | 0 |
|  | ጋሪ? |  | 1 | 0 |
|  | መኪና? |  | 1 | 0 |
| 208 | ዋነኛውየገቢምንጭምንድንነው? | 1 | ግብርና | |
|  |  | 2 | ወርሃዊደመወዝ | |
|  |  | 3 | ንግድ | |
|  |  | 4 | የቤተሰብድጋፍ | |
|  |  | 5 | የዕለትተዕለትሠራተኛ | |
|  |  | 6 | ሌላ | |

3.የጽንስና ጤናአገልግሎትአጠቃቀምተዛማጅመጠይቅ

| ተ.ቁጥር | ጥያቄዎች | የመልስአማራጮች |  |
| --- | --- | --- | --- |
| 301 | ስንትልጅወልደሻል |  |  |
| 302. | ለአሁኑልጅዎነፍሰጡርበነበሩበትጊዜየነፍሴጡርክትትልላይነበሩ? | 1. አዎ 2. 2. አይ | መልስአይከሆነወደጥያቄቁጥር 304 ተሻገር |
| 303 | አዎከሆነስንትጊዜ? | -------------------------- |  |
| 304. | በቅርብየወለድሽውልጅየውልደትቦታየትነበር? | 1. ቤት 2. ጤናጣቢያ 3. የመንግስትሆስፒታል 4. የግልሆስፒታል 5. ሌሎች(ይግለፁ)____________ |  |
| 305. | አሁንላለውልጅከወለዱበኋላየድህረወሊድክትትልአድርገዋል? | 1. አዎ 2. አይ | መልስአይከሆነወደጥያቄቁጥር 401 ተሻገር |
| 306. | አዎከሆነስንትጊዜ? | ____________ |  |

4. የእናትእውቀትደረጃመጠይቅ

| ተ.ቁጥር | ጥያቄዎች | የመልስአማራጮች |  |
| --- | --- | --- | --- |
| 401 | የጨቅላህጸናትየእትብትክብካቤየእውቀትደረጃ |  |  |
| 401.1 | የህፃኑተንከባካቢያለምንምልብስ ፣ ማሰርእናማሰሪያሳይለብስጉቶውንአየርላይማጋለጥአለበት፡፡ | 1. አዎ  2. አይ |  |
| 401.2 | የህፃኑተንከባካቢከሽንትእናከቆሻሻለመከላከልሁልጊዜዳይፐርጠርዞችንከእትብቱበታችማጠፍአለበት | 1. አዎ  2. አይ |  |
| 401.3 | የህፃኑተንከባካቢከተወለደ 24 ሰአትበኋላየተወለደውንልጅማጠብአለበት | 1. አዎ  2. አይ |  |
| 401,4 | የህፃኑተንከባካቢእትብትማጠብያለበትከቆሸሸብቻነው | 1. አዎ  2. አይ |  |
| 401.5 | የህፃኑተንከባካቢየተወለደውንህፃንእትብትከመንከባከብበፊትእናበኋላእጁንመታጠብአለበት | 1. አዎ  2. አይ |  |
| 401.6 | የህፃኑተንከባካቢየተወለደውንህፃንእትብትለመንከባከብምንአይነትየውሃምንጭመጠቀምአለበት | 1. የእጅመታጠቢያውስጥ 2. ወራጅውሃ |  |
| 402. | በቤትውስጥ (የሕፃንስም) እምብርትእንዴትእንደሚንከባከቡመረጃአልዎት? | 1. አዎ  2. አይ | መልስአይከሆነወደጥያቄቁጥር 501 ተሻገር |
| 403. | አዎከሆነ ፣ስለአራስእንክብካቤየመረጃምንጭይጥቀሱ(ከአንድበላይመልስማግኘትይቻላል) | 1. ቤተሰብ/ዘመዶች  2. ጎረቤቶች  3. የጤናባለሙያ  4. ቲቪ/ሬዲዮ  5. ጋዜጣ / መጽሔቶች  6. ሌሎች (ይጥቀሱ) |  |

5. የዕትብትእንክብካቤልምዶችመጠይቅ

| 501 | የህጻኑዕትብትከተቆረጠበሀላምታደረጊበትነገርአለ? | 1. አዎ  2. አይደለም |  |
| --- | --- | --- | --- |
| 502 | አዎከሆነምንድነውምታደርጊበት? | 1. ቅቤ  2. ቫዝሊን  3. ቅባት/ዘይት  4. ሌሎች( ይግለጹ)________ |  |

## ANNEX VI: ጥልቅየቃለመጠይቅመመሪያ

| - 1. ኮድ:   2. የእናትዕድሜ:   3. የመኖሪያቦታ:   4. ሀይማኖት:   5. የት/ትደረጃ:   6. ሥራ:   7. የተወለዱልጆችብዛት:   8. የውልደትቦታ:   9. የህፃኑፆታ: | - 1. የቃለመጠይቅቀን:   2. ቃለመጠይቅየጀመረበትሰዓት:   3. ቃለመጠይቅየተጠናቀቀበትሰዓት:   4. የቃለመጠይቅኮድ:   5. የቴፕቀረፃቁጥር: |
| --- | --- |

**1. አጠቃላይአዲስየተወለዱህፃናትእንክብካቤ**

**1.1** ለልጅዎበህይወትየመጀመሪያወርጤናማእንድሆንያደረጎቸውነገሮችምንድናቸው?

**2 .የእትብትእንክብካቤ**

**2.1.** እባክዎንአዲስለተወለዱህፃናትየዕትብትጉቶበቤትውስጥእንዴትእንደሚንከባከቡይንገሩኝ? እንክብካቤውንስየሰጠውማንነው?

**2.2.** የዕትብትጉቶውንለመንከባከብምክርየሰጠውማንነው? ምንአይነትምክርሰጡ?

መፈተሻ

- የቤተሰብአባላት, የጤናባለሞያዎችወይምሌሎችየማህበረሰብአካላት

**2.3**. በአራስህፃናትየዕትብትጉቶዎላይበቤትውስጥማንኛውምየተደረገነገርነበር? ለምንተደረገ? ካላደረጉምምክንያት?

**2.4**. በማህበረሰብዎውስጥልጆችከተወለዱበሓላበዕትብትጉቶላይበብዛትየሚተገበሩንጥረነገሮችምደናቸው?

**2.5**. ሰዎችይህንንጥረነገርከየትያገኛሉ? መቼነውማድረግየሚጀምሩት? በምንያህልግዜ? ማንውያደረገው? እንዴትነበርየተደረገው? በቀሪውአካልላይየተደረገነገርነበር?

**2.6**. በዕትብትላይየሚደረገውንጥረነገርምንአይነትባህሪያትንይወዳሉ? ምንአይነትስይጠላሉ?

**2.7.** እንዴትበመጀመሪያዎቹቀናትውስጥየዕትብትጉቶላይሌላእንክብካቤሊደረግለትይገባል? በዕትብትጉቶላይካለውንጥረነገርአተገባበርጋርሰላለውግንኙነትይጠይቁ

መፈተሻ:

- ንጽህና
- ቶሎእንድድን
- የዕትብትጉቶውቶሎእንድበጠስ
- የዕትብትጉቶውደርቆእንዲቆይማድረግ
  1. በመጀመሪያዎቹሳምንታትውስጥከዕትብትጉቶጋርተያይዞያሉአደጋዎችምንድናቸው? ሰዎችምንምንለመከላከልእናምላሽለመስጠትነውበዕትብትላይንጥረነገርየሚጠቀሙት

መፈተሻ:

- ቁስል
- ደምመፍሰስ
- ስንጥቅ
