# Supplementary material for: Harmful newborn cord care practices and associated factors among mothers who gave birth in the last six months in Chencha town, Southern Ethiopia: a mixed-methods study
Source: Front Pediatr. 2025 Jan 28;12:1492222. doi: 10.3389/fped.2024.1492222 (PMC11810915; doi:10.3389/fped.2024.1492222)
Supplement: Supplementary file 1 [file Datasheet1.zip › supplementary/annex 1.DOCX]

## ANNEX I: Information Sheet and consent form

**Participant information sheet and informed consent form for individual respondents**

**Introduction**

Good morning/afternoon!

My name is-----------------I am working as data collector for the study being conducted in this town by **Mr. Misgana Seifu** who is studying his master’s degree at Arba Minch University Collage of Health Science and Medicine School of public health Department of MPH-RH postgraduate study. I kindly request you to lend me your attention to explain you about the study and how you have been selected as study participant.

**Study title**– Newborn cord care malpractice and associated factors, among mothers, who gave birth in the last six months in Chencha town, Southern Ethiopia, 2023**.**

**Purpose--** To assess new-born cord care malpractice and associated factors, among mothers, who gave birth in the last six months in Chencha town, Southern Ethiopia, 2023**.**

**Time duration:** The qualified and trained data collectors were collect the necessary information from respondents using an interview guide to have pertinent data that is helpful for the study. The questions will be asked about four major parts, such as socio-demographic and economic related factors, Obstetric and Health service utilization related factors and Mother’s knowledge factors, and Cord care practices questionnaire related characteristics. The content of the interview will be analyzed by the research team and will not be shared with other people. The duration of the interview will be takes time 30-35 minute’s.

**Risks:** The risks of being participating in this study are very minimal, only taking your few minutes.

**Confidentiality:** The information acquired from you will be confidential. There will be no information that will identify in particular. At this moment you may not get any direct benefit by being involved in this study but the information you provide is very important to solve problems on newborn cord malpractice care issue. The data extraction tools will be coded to exclude showing names and other personal information’s. No reference will be made in oral or written reports that could link participants to the study. I would again like to assure you that your answers will be kept confidential, and that you do not have to answer any questions that you do not want to. May I continue?”

**Rights:** Participation in this study is fully voluntary. You have the right to declare not to participate in this study and you have the right to with draw from participating at any time.

**Contact address:** If there is any questions or unclear idea any time about the study or the procedures, do not hesitate to contact and speak to Principal investigator With Cell phone number: **+251916854615** or e-mail address [misganaseifu34@gmail.com](mailto:misganaseifu34@gmail.com), any problem and complain can be address to department public health Arba Minch University, Arba Minch.

**Consent form**

I have read this form or it has been read to me in the language I comprehend and understand all condition stated above.

*Name_____________________________Signature______________________& Date______________________*

*If you do not consent to participate, you do not need to sign this form. Simply you are not obligated to answer.*

*Signature of Investigator _______________________ Date________________________*
