# Supplementary material for: Harmful newborn cord care practices and associated factors among mothers who gave birth in the last six months in Chencha town, Southern Ethiopia: a mixed-methods study
Source: Front Pediatr. 2025 Jan 28;12:1492222. doi: 10.3389/fped.2024.1492222 (PMC11810915; doi:10.3389/fped.2024.1492222)
Supplement: Supplementary file 1 [file Datasheet1.zip › supplementary/annex2.DOCX]

## ANNEX III: ENGLISH VERSION QUESTIONNAIRE

| Description | Identification |
| --- | --- |
| Region | SNNPR |
| Zone | Gamo zone Arbaminch |
| Woreda | Chencha town |
| Kebele |  |
| Supervisor name and signature |  |
| Data collector name and signature |  |
| I.D number/House number |  |
| Date of the interview | **_____/_____/2023** |

**Part I: Socio-demographic and economic questionnaire**

| **S.no.** | **Question** | **Answers and codes** | **Remark** |
| --- | --- | --- | --- |
|  | How old are you? | __________In Years |  |
|  | What is your religion? | 1. Protestant  2. Orthodox  3. Muslim  4. Catholic  5.Others(specify)__________ |  |
|  | What is the **highest level of education** you have completed? | 1. No formal education  2. Primary  3. Secondary  4. College and above |  |
|  | Which of the following best describes your **main work** status over the past 12 months? | 1. House wife  2. Merchant  3. Government employee  4. Self-Employee  5. Student  6.Others(specify)_____________ |  |
|  | Where is your usual place of residence | Urban………………………………1  Rural………………….……………2 |  |
|  | What is your marital status? | 1. Never married 2. Currently married 3. Separated 4. Divorced 5. Widowed 6. Cohabitating |  |
|  | What is your husband’s educational level? | 1. No formal education  2. Primary  3. Secondary  4. College and above |  |
|  | Which of the following best describes your husband’s **main work** status over the past 12 months? | 1. Merchant 2. Government employee 3. Self-Employee 4. Student 5. Others(specify)_____________ |  |
|  | What is your baby’s sex | Male………………………………1  Female……………….……………2 |  |
|  | How old is (baby’s name) now? | __________in days |  |

**Part II: Wealth index questionnaire**

| S.no | Question | Answers and codes | **Remark** |
| --- | --- | --- | --- |
|  |  |  |  |
|  | Who is the owner of the house? | 1. Me 2. Rental 3. Family 4. Other |  |
|  | What is the main material of the roof in your house? | 1. Corrugated sheet 2. Grass 3. Plastic sheet 4. Corrugated iron 5. Other |  |
|  | What is the main material of the dwelling floor in your house? | 1. Soil/sand 2. Wood 3. Ceramic tiles 4. Cement 5. Stone 6. Other |  |
|  | What is the main material of the exterior wall in your house? | 1. Soil/sand 2. Bricks 3. Cement blocks 4. Stone 5. Wood 6. Other |  |
|  | How many rooms are available in this house? | ………………. |  |
|  | How many rooms in this house are used for sleeping? | …………….. |  |
|  | What is the main source of drinking water for member of your household? | 1. Public tap stand 2. Piped to neighbor 3. Piped to yard/plot 4. Piped to dwelling 5. Bottle water |  |
|  | Do you have a separate kitchen | 1. Yes 2. No |  |
|  | What type of toilet facility does this household use? | 1. Water flush latrine 2. Ventilated improved pit latrine 3. No latrine 4. Other …………. |  |
|  | Dose the household have electric power? | 1. Yes 2. No |  |
|  | What type of fuel dose your households mainly use for cooking? | 1. Electricity 2. Wood 3. Charcoal 4. Biogas 5. Natural 6. Other |  |
|  | Does your household have the following materials? | 1. Fixed phone 2. Refrigerator 3. Radio 4. Television 5. Electric mitad 6. Modern bed 7. Other |  |
|  | Does any member of the household have the following resource? | 1. Bicycle 2. Bajaj 3. Motor cycle 4. Car 5. Gari 6. Other |  |
|  | Does any member of the household have mobile phone? | 1. Smart phone 2. Not smart 3. Not |  |
|  | What is the main source of income for the household? | 1. Agriculture 2. Monthly salary 3. Trade 4. Family support 5. Daily laborer 6. Other |  |
|  | Does any member of this house hold have a bank or microfinance saving account? | 1. Yes 2. No |  |

**Part III: Obstetric and Health service utilization related questionnaire**

| S.no | Question | Answers and codes | **Remark** |
| --- | --- | --- | --- |
|  | Parity |  |  |
|  | Have you been on ANC follow up when you were pregnant for your current baby? | 1. Yes  2. No | If no go to  Ques 304 |
|  | If yes, how many times? | _______________ |  |
|  | Where was your place of current delivery? | 1.Home  2. Health center  3. Government Hospital  4. Private Hospital  5.Others(specify)____________ |  |
|  | Have you been on postnatal follow up after birth for your current baby? | 1. Yes  2. No | If no go to  Ques 401 |
|  | If yes, how many times? | _______________ |  |

**Part IV: Mother’s knowledge questionnaire**

| S.no | Question | Answers and codes | **Remark** |
| --- | --- | --- | --- |
|  | Level of knowledge of cord care |  |  |
|  | A care giver is supposed to expose the umbilical stump to air without any dressing, bindings or bandages. | 1. No 2. Yes |  |
|  | A care giver is supposed to folding diaper edges down below the umbilical stump to keep it from urine and soiling all time. | 1. No 2. Yes |  |
|  | A care giver is supposed to wash a newborn after 24 hours of birth | 1. No 2. Yes |  |
|  | A care giver is supposed to wash the cord only if its soiled | 1. No 2. Yes |  |
|  | A care giver is supposed to wash their hand before and after caring for a newborn umbilical stump | 1. No 2. Yes |  |
|  | What source of water a care giver is supposed to use for hand washing in caring for a newborn umbilical stump | 1. Water in basin 2. Running water |  |
|  | Have you had information about how to care for (baby’s name) umbilical cord at home? | 1. No 2. Yes | If no go to  Ques 501 |
|  | If yes, mention the source of information about newborn care (more than one answer is possible) | 1. Family/relatives 2. Neighbors 3. Health professional 4. TV/Radio 5. Newspaper/magazines 6. Others (specify) |  |

**Part V: Cord care practices questionnaire**

| S.no | Question | Answers and codes | **Remark** |
| --- | --- | --- | --- |
|  | Did you apply anything on the cord after cutting? | 1. Yes  2. No |  |
|  | What did you apply? | 1. Butter  2. Vaseline  3. Ointment/oil  4. Others( specify)________ |  |

##

## ANNEX IV: IN-DEPTH INTERVIEW GUIDE

| - 1. ID:   2. Mother’s age:   3. Place of residence:   4. Religion:   5. Education:   6. Occupation:   7. Total number of children delivered:   8. Place of delivery:   9. Sex of baby: | - 1. Interview date:   2. Interview start time:   3. Interview end time:   4. Interviewer code:   5. Tape recording number: |
| --- | --- |

1. **General care of the newborn**
   1. What are the things that you have done to keep your baby healthy in the first month of life?
2. **Umbilical cord care**
   1. Please tell me how your newborn’s cord stump was cared for at home? Who gave such care?
   2. Who gave advice on caring for the cord stump? What advice did they give?

Probe

- Family members, health workers or other community members
  1. Was anything applied to your newborn’s cord stump at home? Why it was (not) applied?
  2. What are the substances that commonly applied to the cord stump after birth in your community?
  3. Where do people get this substance? When did application start? How frequently? Who applied it? How was it applied? Was anything applied on the rest of the body?
  4. What characteristics of the substance applied on the cord do you like? What characteristics do you dislike?
  5. How else the cord stump should be cared for in the first few days? Inquire about its relation to application of substance on the cord

Probe:

- Cleanliness
- Promotion of healing
- Speeding-up separation
- Maintaining a dry stump
  1. What are the risks related to the cord in the first few weeks? What are people’s efforts to prevent and respond to them in relation to application of substance on the cord.

Probe:

- Sores
- Bleeding
- Cracking
