# Supplementary figures and images for: Harmful newborn cord care practices and associated factors among mothers who gave birth in the last six months in Chencha town, Southern Ethiopia: a mixed-methods study
Source: Front Pediatr. 2025 Jan 28;12:1492222. doi: 10.3389/fped.2024.1492222 (PMC11810915; doi:10.3389/fped.2024.1492222)

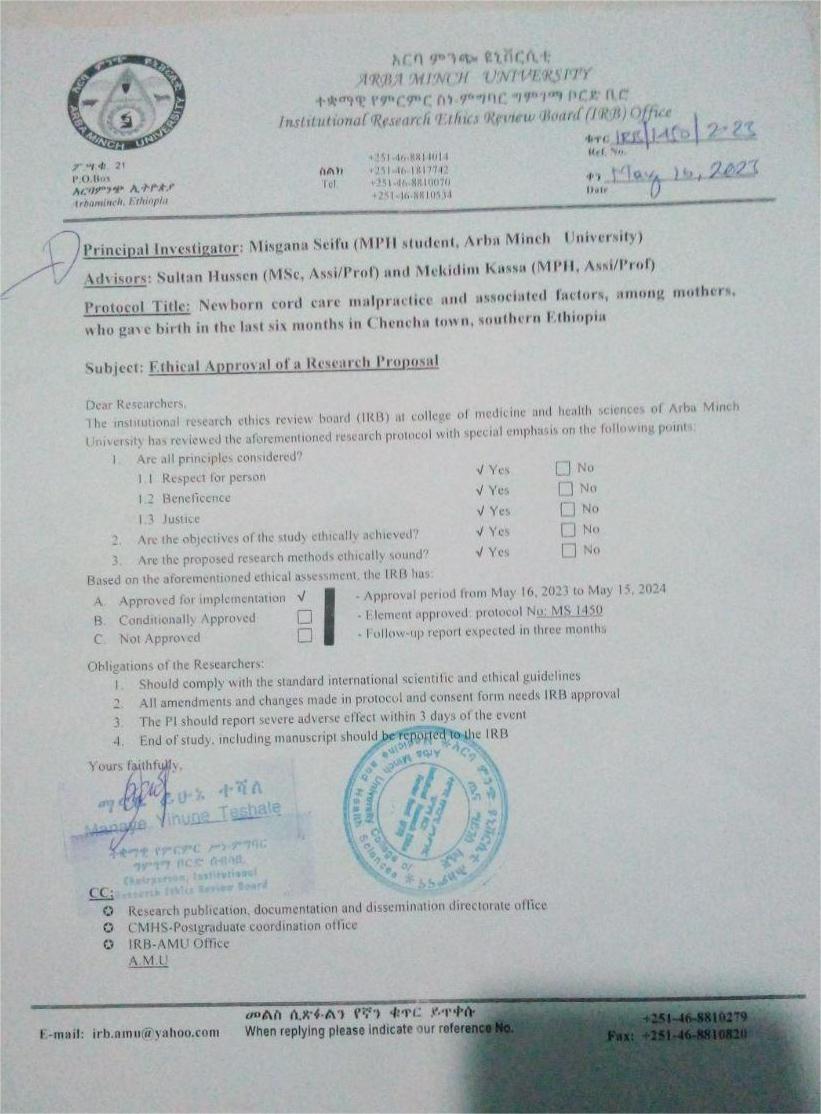

Supplement: Supplementary file 1 [file Datasheet1.zip › supplementary/Image 1.JPEG]
